# Effect of Rosiglitazone and Insulin Combination Therapy on Inflammation Parameters and Adipocytokine Levels in Patients with Type 1 DM

**DOI:** 10.1155/2015/807891

**Published:** 2015-07-27

**Authors:** Metin Guclu, Ozen Oz Gul, Soner Cander, Oguzkaan Unal, Guven Ozkaya, Emre Sarandol, Canan Ersoy

**Affiliations:** ^1^Division of Endocrinology, Sevket Yılmaz Research and Education Hospital, 16310 Bursa, Turkey; ^2^Department of Endocrinology and Metabolism, School of Medicine, Uludağ University, 16210 Bursa, Turkey; ^3^Acibadem Private Hospital, 16210 Bursa, Turkey; ^4^Department of Biostatistics, School of Medicine, Uludağ University, 16210 Bursa, Turkey; ^5^Department of Biochemistry, School of Medicine, Uludağ University, 16210 Bursa, Turkey

## Abstract

*Aim.* To investigate the efficacy of combined therapy of insulin and rosiglitazone on metabolic and inflammatory parameters, insulin sensitivity, and adipocytokine levels in patients with type 1 diabetes mellitus (type 1 DM).* Material and Methods.* A total of 61 adults with type 1 DM were randomly and prospectively assigned in open-label fashion to take insulin and rosiglitazone 4 mg/day (*n* = 30) or insulin alone (*n* = 31) for a period of 18 weeks while undergoing insulin therapy without acute metabolic complications.* Results.* Combination therapy did not significantly improve metabolic and inflammatory parameters, insulin sensitivity, and adiponectin levels. While leptin and resistin levels decreased in both groups (group 1: resistin 6.96 ± 3.06 to 4.99 ± 2.64, *P* = 0.006; leptin 25.8 ± 17.6 to 20.1 ± 12.55, *P* = 0.006; group 2: resistin 7.16 ± 2.30 to 5.57 ± 2.48, *P* = 0.031; leptin 16.72 ± 16.1 to 14.0 ± 13.4, *P* = 0.007) Hgb and fibrinogen levels decreased only in group 1 (Hgb 13.72 ± 1.98 to 13.16 ± 1.98, *P* = 0.015, and fibrinogen 4.00 ± 1.08 to 3.46 ± 0.90, *P* = 0.002). Patients in both groups showed weight gain and the incidence of hypoglycemia was not lower.* Discussion.* The diverse favorable effects of TZDs were not fully experienced in patients with type 1 DM. These results are suggesting that insulin sensitizing and anti-inflammatory characteristics of TZDs were likely to be more pronounced in patients who were not totally devoid of endogenous insulin secretion.

## 1. Introduction

There has been a progressive increase in the incidence of type 1 DM, and several advances in its treatment have been achieved. As a result, an increasing number of patients, who are older and have longer disease durations, are more severely affected by chronic complications [1–3]. Chronic subclinical inflammation, impaired fibrinolytic system activity, and elevated procoagulant factor levels form the basis of atherosclerotic diseases. Consequently, DM has been regarded as a major risk factor for cardiovascular diseases [4, 5]. Insulin resistance is characterized by limited stimulation of glucose metabolism in muscle and the liver and has been described in patients with poorly controlled type 1 DM [6–8].

Fatty tissue releases a number of adipocytokines associated with neuroendocrine and immune functions. Cytokines such as leptin, resistin, and adiponectin released from these tissues critically impact nutritional status, body fat distribution, metabolic parameters, inflammatory status, atherosclerotic alterations, and insulin resistance [9]. The levels of adiponectin, which is regarded as an antidiabetic, anti-inflammatory, and antiatherogenic cytokine, are reported to be depressed in patients with type 2 DM [10, 11]. However, in some studies, increased adiponectin levels have been reported in patients with type 1 DM [12]. Resistin, on the other hand, impairs cellular glucose intake, because it is stimulated by insulin; as a result, hepatic glucose production is increased, leading to impaired glucose tolerance and eventual development of insulin resistance. Owing to its augmenting effect on the production of adhesion molecules, resistin is considered to be having proinflammatory effects in the vascular endothelium [13, 14]. Leptin has been shown to have important effects on both body energy balance and fat distribution [15, 16].

Glucose uptake rates by peripheral tissues, which can be stimulated by insulin in skeletal muscles, decrease over time in patients with type 1 DM and poor glycemic control. These patients have significant hepatic insulin resistance, and the effects of insulin are impaired because of plasma free fatty acids (FFAs) [17–19]. Despite recent advances in the management of DM, it has been suggested that cardiovascular disease-related mortality rates increase as more intensive therapies are required to ensure tight blood glucose control. Therefore, combination therapies are required to ensure tight glycemic control, minimize the risk of macrovascular disease, and reduce other cardiovascular risk factors [20].

Thiazolidinediones (TZDs) act by binding to “nuclear peroxisome proliferator activated receptor-gamma” (PPAR-*γ*), which is chiefly expressed in fatty tissue, and mediate their effects by activating the transcription of the genes that influence adipocyte differentiation as well as glucose and lipid metabolism [21–23]. TZDs decrease the triglyceride concentration in *β*-cells, leading to improved *β*-cell function. TZDs, apart from their direct effect on fatty tissue, might influence the release of adipocyte-derived signal factors that determine the insulin sensitivity of muscles, such as FFAs, adiponectin, leptin, and tumor necrosis factor-alpha (TNF-*α*). In addition to their favorable effects on glycemic control, TZDs directly influence vessel walls, decrease vasoconstriction, and inhibit inflammation. Therefore, they inhibit insulin resistance and slow down the atherosclerotic process [24–27]. In addition, it has been shown that TZDs could inhibit hyperglycemia-induced reactive oxygen species production from mitochondria (mtROS) by activating the PPAR-*γ* coactivator-1 alpha (PGC-1*α*) pathway which could contribute to the prevention of diabetic vascular complications [28, 29]. Agonists of PPAR-gamma and PPAR-alpha have been shown to upregulate the heme-oxygenase- (HO-) system which has been shown to increase insulin sensitivity, improve glucose/lipid metabolism, suppress inflammation/oxidative stress, decrease immune response, and modulate cell-growth/differentiation [30]. It has been also shown that there were beneficial effects of the HO-system in the pathogenesis of type 1 diabetes and related cardiometabolic complications [31].

Although, euglycemic-hyperinsulinemic clamp study is accepted standard for measurement of insulin sensitivity in patients with type 1 DM, it is not practical for use and is labor-intensive. Estimated glucose disposal rate (eGDR) is a derived measure of insulin resistance and can be calculated using routine clinical measures such as waist circumferences or waist-to-hip ratio, presence of hypertension, and HbA1c levels. As an insulin sensitivity index, it is well correlated with results obtained from clamp studies and it should be emphasized that lower eGDR levels indicate greater insulin resistance [32, 33].

The purpose of the present study was to investigate the efficacy of combined therapy of insulin and the oral antihyperglycemic agent rosiglitazone, a PPAR-*γ* agonist, on blood glucose regulation, total administered daily insulin dose, metabolic parameters, eGDR, FFAs, inflammatory indicators, and adipocytokine levels in patients with type 1 DM and poor glycemic control despite intensive insulin therapy.

## 2. Materials and Methods

The study was conducted between March 2007 and January 2008 at the Clinic of Endocrinology and Metabolism Diseases, following the approval of the Uludağ University Medical School Ethics Committee. After providing written consent, the patients were considered eligible based on the following criteria: age 18–65 years, diagnosis of type 1 DM, and a glycosylated hemoglobin (HbA1c) level >6.5% despite 40-unit (U) intensive insulin therapy, on average, for ≥6 months. Exclusion criteria were renal failure (glomerular filtration rate <75 mg/min or creatinine >1.5 mg/dL), chronic hepatic disease or aspartate aminotransferase (AST) and alanine aminotransferase (ALT) levels 2.5 times the normal values, current antidiabetic therapy other than insulin therapy, known history of rosiglitazone allergy, stage II–IV heart failure according to the New York Heart Association (NYHA) classification [34], inability of the patient to enforce strict lifestyle changes, medical nutrition therapy or self-monitoring of blood glucose levels, ongoing or planned pregnancy, and current lactation.

### 2.1. Study Protocol

This prospective, open-label, randomized trial investigated the effectiveness of 4 mg/day rosiglitazone for 18 weeks in patients undergoing insulin therapy and without acute metabolic complications in combination with strict lifestyle changes and effective medical nutrition therapy. The patients attended a screening visit (Visit 1) two weeks before randomization, and they were classified into two open-label groups, such that clinical and demographical characteristics were similar in the two groups. The patients were evaluated for lifestyle changes, diet and exercise compliance, and insulin requirements. Their therapies were modified, and the randomization visit (Visit 2) occurred two weeks later. The patients in both groups underwent insulin titration at Visit 2, and the patients in one group had 4 mg/day rosiglitazone added to their ongoing therapy (group 1), while the patients in the other group were monitored from that point with their most recent insulin titration (group 2). To reduce the risk of side effects, 4 mg rosiglitazone was initially administered in group 1. Then, the patients attended a control visit (Visit 3) four weeks after randomization and a final visit (Visit 4) after 16 weeks. During each of the four visits, variables within six different categories were monitored, including a detailed physical examination; self-monitoring of BG levels; glycemic control; therapy alterations and insulin requirements; adverse events; and biochemical, hematological, and inflammatory parameters, including adipocytokine levels.

### 2.2. Glycemic Control and Insulin Titration

In order to measure the direct effect of rosiglitazone, administered insulin doses were not changed unless patients developed acute metabolic complications. Recurrent hypoglycemia, presence of symptomatic hyperglycemia, diabetic ketosis (DK), diabetic ketoacidosis (DKA), and nonketotic hyperosmolar syndrome (NKHS) were considered metabolic complications. While patients with DK, DKA, NKHS, or major hypoglycemia were planned to be excluded from the study and hospitalized if necessary, patients with symptomatic hyperglycemia and recurrent minor hypoglycemia underwent insulin titration. Patients with symptoms of polydipsia, polyuria, weight loss, and nocturia were considered to be having symptomatic hyperglycemia if they also had mean BG levels >276 mg/dL; minor hypoglycemia if they were aware enough of their condition to administer self-therapy, were symptomatic, and had BG levels <56 mg/dL; or major hypoglycemia if they were unconscious, were unable to treat themselves, and recovered after therapy administered by others at home or at hospital.

### 2.3. Laboratory Methods

Blood samples were analyzed for fasting plasma glucose (FPG), urea, creatinine (Cr), AST, ALT, total cholesterol (total-C), high-density lipoprotein cholesterol (HDL-C), low-density lipoprotein cholesterol (LDL-C), and triglycerides (TG) using an autoanalyzer (Aeroset System Operations Manual; Abbot Laboratories, Abbott Park, IL, USA). LDL-C levels in patients with TG values <400 mg/dL were calculated using the Friedewald formula as follows: LDL-C = total-C − [(TG/5) + HDL-C].

HbA1c levels were measured using high performance liquid chromatography (HPLC; BIO RAD Diagnostic Group, Hercules, CA, USA). Spot urine albumin excretion was measured by chemiluminescence immunoassay (Immulite 2500 Analyzer; Siemens, CA, USA), and creatinine levels were measured spectrophotometrically (Aeroset System Operations Manual; Abbot Laboratories, Abbott Park, IL, USA); in addition, the albumin/creatinine ratio (ACR) was calculated.

The eGDR was calculated as follows: 21.158 + (−0.009 × WC) + (−3.407 × HTN) + (−0.51 × HbA1c), where the WC indicates the waist circumferences and HTN indicates blood pressure and is expressed as 0: no, 1: yes. The range of clamp-measured glucose disposal rates in the eGDR validation study was 3.8 to 13.4, with a range of ~9 to 11 in those with normal insulin resistance [32].

FFA measurements were conducted using Wako NEFA-HR [2] (Wako Chemicals, GmbH, Neuss, Germany) and an* in vitro* enzymatic calorimetric assay method. The measureable range was 0.01–4.00 mEq/L.

White blood cell (WBC), hemoglobin (Hgb), hematocrit (Hct), and platelet (PLT) measurements were conducted using Cell-Dyn 3700 (MAPSS Laser Differential; Abbott Laboratories, Abbott Park, IL, USA).

Blood samples were collected as indicated and placed in Westergren tubes to establish erythrocyte sedimentation rates (ESRs) in a 1-hour period. The results were recorded as ESR in mm/h.

Fibrinogen was measured using the Coagulation System, Dade Behring BNII (Dade Behring Inc., Marburg, Germany). The measurable range was <1.8–3.5 mg/L.

The solid phase enzyme linked immunosorbent assay (ELISA) method was used with a High Sensitivity CRP Enzyme Immunoassay (DRG International Inc., Mountainside, NJ, USA) for the measurement of high-sensitivity C-reactive protein (hs-CRP). The measurable range was <0.1–10 mg/L, and intra- and interassay coefficients were 2.5% and 2.3%, respectively.

The solid phase ELISA method and a DRG Leptin Enzyme Immunoassay Kit (DRG GmbH, Marburg, Germany) were used to measure leptin. The expected results were 3.84 ± 1.79 ng/mL for men and 7.36 ± 3.73 ng/mL for women. The intra- and interassay coefficients were 5.95% and 8.66%, respectively.

Resistin was measured using the ELISA method with Human Resistin ELISA (BioVendor, Brno, Czech Republic). The normal range was considered to be 8.1 ± 4.0 ng/mL. The intra- and interassay coefficients were 2.8% and 5.1%, respectively.

Adiponectin was measured using the ELISA method with a High Sensitivity Human Adiponectin ELISA kit (BioVendor, Brno, Czech Republic). The normal range was dependent upon BMI and was 9.5 ± 3.9 *μ*g/mL for men and 13.2 ± 6.1 *μ*g/mL for women. The intra- and interassay coefficients were 4.1% and 4.0%, respectively.

### 2.4. Statistical Analysis

According to pilot study, a sample size of 30 patients per group was calculated to give 80% power to detect a difference of 6 in change from baseline in mean adiponectin between groups at the 5% 2-sided significance level, assuming 8 as the common SD. Statistical analyses were conducted using SPSS for Windows (Version 22.0; SPSS Inc., Chicago, IL, USA). Continuous variables are expressed as the mean ± standard deviation or median (minimum-maximum), as appropriate; categorical variables are expressed as frequencies (*n*, %). A one-way ANOVA was used to compare mean values for normally distributed variables when there were more than two independent groups, and the Kruskal-Wallis test was used when the assumptions for parametric tests were not met. The Mann-Whitney *U* test was used for nonparametric comparison of two groups. Paired data were analyzed using paired *t*-test and the Wilcoxon signed rank test when data were not normally distributed. For measurement at last visit, percent changes were calculated according to baseline measurement. These percent changes were compared. Pearson's chi-squared was used for comparison of the frequencies. *P* < 0.05 was considered statistically significant in all tests.

## 3. Results

The demographic characteristics and baseline glycemic levels are shown in Table 1.

There were no significant differences between the groups in sex, mean age, disease duration, BW, BMI, waist circumferences, waist-to-hip ratios, FPG, HbA1c values, and eGDR in the baseline and mean *P* value was higher than 0.05 for all parameters mentioned above. eGDR was 8.53 ± 2.70 mg/kg/min in group 1 and 9.25 ± 3.18 mg/kg/min in group 2. Patients in group 1 had higher FPG and HbA1c and slightly low eGDR, in other words more insulin resistant than group 2.

The comparisons between the final and baseline values for each group are shown in Table 2.

The total number of patients in each group that completed the study was 28, as two patients in each group were excluded owing to acute metabolic complications and one patient in group 2 was excluded owing to noncompliance with the visit schedule. During the follow-up period, the changes in BW and BMI were statistically significant in both groups (*P* < 0.05 for group 1 and *P* < 0.01 for group 2), whereas the changes in WC and waist-to-hip ratio (WHR) were not significant.

Systolic blood pressure (SBP) significantly decreased in group 2; the changes in diastolic blood pressure (DBP) were similar in both groups. Although the final FPG and HbA1c values were lower than the baseline values in both groups, the changes were statistically significant only in group 2. No statistically significant differences were found in eGDR during the study period in both groups. eGDR was 8.53 ± 2.70 mg/kg/min at baseline and 8.36 ± 2.45 mg/kg/min at final visits in group 1. It was 9.25 ± 3.18 mg/kg/min at baseline and 9.22 ± 3.20 at final visits in group 2. *P* value was 0.185 for group 1 and 0.235 for group 2 in terms of eGDR changes between two visits. Urea, Cr, spot urine ACR, AST, and ALT levels did not significantly change in either group. Of the total-C, HDL-C, LDL-C, TG, and FFA levels, only the HDL-C levels in group 2 significantly changed (*P* = 0.038).

While Hgb and HCT values did not change significantly in group 2, a significant decrease in Hgb levels was observed in group 1. Of the ESR, fibrinogen, and hs-CRP levels, only fibrinogen levels significantly decreased in group 1, and the changes in the other parameters were not significant for either group.

Although the resistin and leptin levels decreased significantly and changes were statistically significant in both groups, adiponectin levels increased but the change was not significant. The total daily insulin doses administered during the follow-up period are also shown in Table 2. The changes observed both between and within the groups in total daily insulin doses per kilogram of BW were not significant.

Comparisons between the groups for the final and baseline values of inflammatory markers, adipocytokine, and FFA levels are shown in Table 3.

The differences between the groups in ESR and hs-CRP levels were not significant at both baseline and final visits. However, while the difference in fibrinogen levels was significant at the baseline visit, the values at the final visit were not different owing to the decrease in fibrinogen levels in group 1. Also, the differences between the groups in resistin, leptin, and adiponectin levels were not significant at both baseline and final visits.

Diabetes-related complications experienced by the patients over the 18-week follow-up period are shown in Table 4.

The mean hypoglycemia frequency was calculated by dividing the number of total hypoglycemia episodes for all of the patients over the 18-week period by the number of patients, and the mean change in BW for the same period was calculated similarly. The number of patients with acute complications or major hypoglycemic events was expressed as the total frequency for the group. Two patients in group 1 experienced major hypoglycemic events, while two patients in group 2 had to be hospitalized due to DKA (Table 4). While 5.35 minor hypoglycemia episodes were experienced per patient over the 18 weeks in group 1, the mean weight gain was 2.58 ± 3.10 kg during the same period. The patients in group 2, on the other hand, experienced 4.61 minor hypoglycemic episodes per patient, and the mean weight gain was 1.47 ± 1.53 kg during the same period. The *P* value for minor hypoglycemia was 0.437, and it was 0.142 for weight gain. Therefore, the difference between the groups with respect to either parameter was not statistically significant. Because of the low number of major hypoglycemia events and acute metabolic complications, statistical comparisons were not possible.

## 4. Discussion

The combined therapy approaches used in type 2 diabetes management have also become increasingly common in type 1 diabetes management. Furthermore, studies have demonstrated that insulin resistance is not exclusively observed in patients with type 2 diabetes and that it is also a critical factor for patients with type 1 diabetes because it can be overlooked in patients without adequate BG regulation [32, 35–38]. The primary goals when introducing insulin-sensitizing agents in combined therapy are ensuring good glycemic control, decreasing insulin demand, achieving favorable effects on cardiovascular risk factors, and minimizing alterations in BW. Strowig and Raskin reported improved glycemic control without an increase in insulin demand in 25 overweight adult patients with type 1 diabetes following therapy with 4 mg rosiglitazone twice a day for 8 months [39]. Although patients in both groups in the present study had decreased FPG and HbA1c levels, the changes were not significant. Baseline glucose and HbA1c values were higher in the patients receiving rosiglitazone; however, the differences in the changes between the groups were not significant. In a study conducted by Stone et al. [40], 36 patients with type 1 diabetes whose daily insulin requirement was >1.1 U/kg were administered 8 mg/day rosiglitazone. The investigators reported that the change in HbA1c levels was not significant when compared with the placebo group.

For decades, type 1 diabetes has been traditionally known as insulin-dependent, while type 2 has been known as noninsulin-dependent diabetes. However, it is becoming increasingly clear that insulin deficiency and insulin resistance are manifested in both forms of diabetes at different stages. Unlike other studies [39, 40] which investigate the effects of TZDs on insulin resistance and consist of obese or overweight patients with type 1 DM, almost all of the patients were lean in present study. eGDR is a validated clinical tool for estimating insulin sensitivity in patients with type 1 DM. It was near normal at baseline and did not change significantly during our study period in both groups. This result was important to show effects of rosiglitazone, except insulin-sensitizing characteristics. Thus, it can be thought that the effects of rosiglitazone observed in present study were not associated with insulin sensitivity. One of the known major effects of these drugs is on oxidative stress and mitochondrial ROS production. These effects may be key factors during the development of diabetic vascular complications. It has been shown that TZDs could inhibit hyperglycemia-induced mtROS and contribute to the prevention of diabetic vascular complications [41]. Although there were some studies investigating these effects in patients with type 2 DM, it is unclear in patients with type 1 DM. It is obvious that there is a need to investigate this effect in prospective cohort studies composed of patients with type 1 DM. In addition, agonists of PPAR-gamma might increase insulin sensitivity via upregulating heme-oxygenase-system. The HO-system and related products have been shown to decrease inflammation and enhance insulin sensitivity. More importantly, in experimental models of type 1 DM, upregulating HO-system caused increase in pancreatic beta cell insulin production. Beneficial effects of TZDs on insulin resistance and inflammation resulting from HO-system have been studied in patients with both type 2 and type 1 DM [30, 31, 42]. These developments may offer new options, either prevention of disease or development of the complications.

We observed significant BW and BMI changes in all patients at the final visit compared with baseline values. However, weight gain did not significantly differ between the baseline and final visits. The most significant side effect of TZDs, particularly when combined with sulfonylurea and insulin, is weight gain. TZDs increase overall fat tissue, and the most affected area is subcutaneous fat tissue. Another major cause of TZD-related weight gain is increased water and salt retention, which leads to increased plasma volume. Consequently, these drugs might lead to complications in patients with heart failure. Edema is caused by depressed renal sodium excretion and free water retention [43–45]. Sotton et al. reported a 10% increase in left ventricular mass without significant alterations in cardiac structure and function in patients undergoing rosiglitazone therapy. They attributed it mainly to the increase in plasma volume [46]. Although we observed weight gain in our patient groups in this present study, no patient had heart failure. Moreover, peripheral edema or dyspnea was not among the adverse events reported by our patients. This could be attributed to the relatively young mean age in our sample or to the fact that heart failure at baseline was among the exclusion criteria. Furthermore, no patient in this cohort had diabetic nephropathy that could lead to edema, which might have decreased the risk for edema.

The hypoglycemic effects of TZDs include increased insulin sensitivity that is mediated through TG and FFA metabolism and is associated with the agonistic effects of PPAR-*γ*. Increased FFA levels lead to insulin resistance and fasting plasma FFA levels in patients with type 2 diabetes administered TZD might decrease by 20–30%. PPAR-*γ* activation impairs TG and fatty acid synthesis, leading to decreased very-LDL-C and HDL-C synthesis as well as increased LDL-C and total-C levels [47, 48]. The patients in the present study with BMI and WC levels in the normal range had baseline TG and HDL-C levels within the desired range, and the LDL-C levels in all patients were close to 100 mg/dL. While slight decreases in total-C, LDL-C, TG, and FFA levels were noted in the patients receiving rosiglitazone at the end of the present study, HDL-C levels had increased slightly. Patients receiving only insulin had similar values to those in the combined therapy group except for slightly higher TG levels, although this was not statistically significant. Comparisons within and between the groups did not show significant differences. We suggest that the strict enforcement of lifestyle changes during the follow-up period might have contributed to these results.

TZDs have been demonstrated to have significant anti-inflammatory characteristics in studies conducted on patients with type 2 diabetes. Calkin et al. reported that rosiglitazone reduces diabetes-related atherosclerosis and that this effect is possibly associated with oxidative stress and inflammation, independent of metabolic effects, unrelated to the insulin dose [49]. In particular, the Diabetes Control and Complications Trial, as well as a number of other studies, revealed that hs-CRP levels increased in patients undergoing intensive therapy. Furthermore, hs-CRP and increased fibrinogen levels are independent risk factors for coronary heart disease, and a number of studies have reported elevated fibrinogen levels in patients with diabetes [50, 51]. The ESR levels in the present study were within the normal range at baseline, and they continued to be so until the end of study. While hs-CRP levels decreased with respect to baseline values in the patients receiving rosiglitazone in the present study, they increased slightly in the patients receiving insulin alone. To the best of our knowledge, this is the first study to measure fibrinogen levels in patients with type 1 diabetes after rosiglitazone administration. The levels decreased significantly in these patients, while the patients undergoing therapy with insulin alone showed minimal decreases in fibrinogen levels. Independent of the improvement in BG regulation, levels of hs-CRP and fibrinogen, which are conventional inflammatory markers, decreased significantly following rosiglitazone therapy in the present study.

Plasma leptin levels are positively correlated with female sex, BMI, and age but not with diabetes duration, HbA1c, or total insulin dose per kilogram [52]. Most patients with type 1 diabetes are either underweight or in the normal BW range. Data concerning leptin levels in this group of patients vary, and exogenous insulin therapy leads to elevated leptin levels in patients with type 1 diabetes [53, 54]. Resistin, which is released from adipose tissue, is directly associated with insulin resistance factors such as WC and WHR. Shalev et al. reported increased serum resistin levels in patients with type 1 diabetes and reported that levels returned to the normal range after pancreas transplant [55, 56]. The patients in the present study had normal BW, BMI, and WC. The leptin levels were within the normal range at both baseline and final visits. While no significant differences were observed between the groups at the baseline or final visit or in the change between the visits, the within-group changes during the follow-up period were significant, despite the weight gain. Resistin levels were low in both groups, and the change in the patients receiving rosiglitazone was significant, despite weight gain, and was associated with the favorable effects of rosiglitazone.

Adiponectin has been shown to have positive effects on cardiometabolic risk, and adiponectin levels are negatively correlated with insulin resistance and weight gain. Moreover, good glycemic control increases adiponectin levels, whereas poor glycemic control decreases adiponectin levels [57, 58]. In the CACTI trial, Maahs et al. reported negative correlations between adiponectin levels and male sex, central adiposity, SBP, DBP, daily insulin dose, HbA1c, fibrinogen, albumin excretion rate, and TG levels; positive correlations were noted with type 1 diabetes, HDL-C, and homocysteine [59]. In the present study, adiponectin levels increased parallel to glycemic improvement both in patients undergoing combined therapy and in those receiving insulin therapies alone. Thus, the increase was independent of the therapy.

In conclusion, the diverse favorable effects of TZDs previously reported in patients with type 2 diabetes were not fully experienced in patients with type 1 diabetes in the present study. The addition of 4 mg/day rosiglitazone to intensive insulin therapy did not significantly improve glycemic parameter, lipid parameter, FFA, ESR, hs-CRP, leptin, or resistin levels. Only fibrinogen levels were significantly different between the groups. Patients receiving rosiglitazone showed weight gain, a major side effect of TZDs, whereas insulin sensitivity was not significantly different and the incidence of hypoglycemia was not lower. HbA1c, FPG, and total daily insulin doses decreased significantly after combination therapy with TZD and insulin in patients with type 2 diabetes mellitus, suggesting that the insulin-sensitizing characteristics of TZDs are likely more pronounced in patients who are not totally devoid of endogenous insulin secretion.

We acknowledge the limitations of the current study including single centre experience and small sample size. Patients treated in our series did not appear to have driven any meaningful benefit from combination therapy. Due to small number of patients included in study, prospective clinical trials are however required to define optimal treatment regimens. Clinical prognostic factors evaluated in our series may be useful for stratification and eligibility considerations in future clinical trials.

## Figures and Tables

**Table 1:** Comparison of the demographic characteristics and baseline glycemic parameters between the groups.

Characteristics	Group 1 Insulin + Ros (*n* = 30)	Group 2 Insulin alone (*n* = 31)	*P*
Sex (women/men)	18/12	17/14	0.570
Age (years)	27.55 ± 8.48	27.09 ± 5.38	0.734
Diabetes duration (years)	10 ± 4.95	9.6 ± 4.92	0.814
BW (kg)	66.59 ± 8.6	63.13 ± 8.2	0.768
BMI (kg/m^2^)	24.17 ± 2.62	22.97 ± 2.74	0.606
WC (cm)	83.80 ± 8.26	78.71 ± 9.11	0.135
WHR	0.85 ± 0.06	0.80 ± 0.06	0.773
FPG (mg/dL)	249.1 ± 69.5	223.2 ± 78.5	0.167
HbA1c (%)	9.22 ± 1.77	8.75 ± 1.14	0.886
eGDR (mg/kg/min)	8.53 ± 2.70	9.25 ± 3.18	0.120

BW: body weight; BMI: body mass index; WC: waist circumference; WHR: waist-to-hip ratio; SBP: systolic blood pressure; DBP: diastolic blood pressure; FPG: fasting plasma glucose; HbA1c: glycosylated hemoglobin; eGDR: estimated glucose disposal rate.

**Table 2:** Comparison between the baseline and final (week 16) values for each group.

Parameters	Group 1 Insulin + rosiglitazone			Group 2 Insulin alone		
	Baseline (*n* = 30)	Final (*n* = 28)	*P*	Baseline (*n* = 31)	Final (*n* = 28)	*P*
BW (kg)	66.59 ± 8.6	69.96 ± 9.29	**0.003**	63.13 ± 8.2	65.18 ± 8.20	**0.001**
BMI (kg/m^2^)	24.17 ± 2.62	25.69 ± 2.57	**0.002**	22.97 ± 2.74	23.76 ± 2.73	**<0.001**
WC (cm)	83.80 ± 8.26	86.61 ± 8.53	0.107	78.71 ± 9.11	80.78 ± 8.09	0.109
WHR	0.85 ± 0.06	0.85 ± 0.05	0.792	0.80 ± 0.06	0.82 ± 0.06	**0.048**
SBP (mmHg)	114 ± 13.63	111.6 ± 13.7	0.604	116 ± 9.37	110.5 ± 9.62	**0.030**
DBP (mmHg)	75.25 ± 9.24	70.83 ± 9.11	0.061	75.95 ± 7.51	72.63 ± 7.70	0.174
FPG (mg/dL)	249.1 ± 69.5	219.7 ± 97	0.460	223.2 ± 78.5	178 ± 91	**0.006**
HbA1c (%)	9.22 ± 1.77	9.09 ± 1.40	0.239	8.75 ± 1.14	8.46 ± 1.19	0.050
eGDR (mg/kg/min)	8.53 ± 2.70	8.36 ± 2.45	0.185	9.25 ± 3.18	9.22 ± 3.20	0.235
Urea (mg/dL)	25.50 ± 7.17	26.66 ± 6.2	**0.032**	25.52 ± 6.03	27.84 ± 4.96	**0.042**
Cr (mg/dL)	0.83 ± 0.16	0.82 ± 0.26	0.935	0.86 ± 0.12	0.90 ± 0.31	0.602
ALT (IU/L)	18.25 ± 11.4	13.55 ± 4.3	**0.033**	15.85 ± 9.85	17.52 ± 13.3	0.912
Total-C (mg/dL)	172.8 ± 36.2	169.0 ± 31.8	0.935	177.3 ± 33.8	170.1 ± 34.5	0.678
HDL-C (mg/dL)	45 ± 5.90	47.80 ± 9.37	0.285	47.00 ± 9.35	51.47 ± 10.60	**0.038**
LDL-C (mg/dL)	105 ± 27.12	98.86 ± 27.89	0.688	111.15 ± 24.17	107.45 ± 30.1	0.668
TG (mg/dL)	113 ± 77.91	111.20 ± 52.41	0.971	79.47 ± 42.95	81.57 ± 27.14	0.821
FFA (mEq/L)	0.57 ± 0.43	0.53 ± 0.28	0.913	0.49 ± 0.25	0.47 ± 0.26	0.601
ACR (mg/min)	35.50 ± 42.91	23.57 ± 17.65	0.136	19.36 ± 19.00	12.54 ± 10.72	0.667
Hgb (g/dL)	13.72 ± 1.98	13.16 ± 1.98	**0.015**	13.74 ± 1.64	13.61 ± 1.04	0.838
HCT (%)	40.55 ± 5.48	39.06 ± 5.42	0.067	40.53 ± 4.44	40.65 ± 2.69	0.428
Resistin	6.96 ± 3.06	4.99 ± 2.64	**0.006**	7.16 ± 2.30	5.57 ± 2.48	**0.031**
Leptin	25.8 ± 17.6	20.1 ± 12.55	**0.006**	16.72 ± 16.1	14.0 ± 13.4	**0.007**
Adiponectin	17.48 ± 10.71	19.81 ± 11.21	0.145	11.90 ± 5.23	15.98 ± 9.47	0.948
Insulin dose (units/day)	64.45 ± 16.31	65.88 ± 15.29	NS	53.33 ± 13.45	52.78 ± 11.07	NS

BW: body weight; BMI: body mass index; WC: waist circumference; WHR: waist-to-hip ratio; SBP: systolic blood pressure; DBP: diastolic blood pressure; FPG: fasting plasma glucose; HbA1c: glycosylated hemoglobin; eGDR: estimated glucose disposal rate; CR: creatinine; AST: aspartate aminotransferase; ALT: alanine aminotransferase; Total-C: total cholesterol; HDL-C: high-density lipoprotein cholesterol; LDL-C: low-density lipoprotein cholesterol; TG: triglycerides; FFA: free fatty acids; ACR: albumin/creatinine ratio; Hgb: hemoglobin; Hct: hematocrit.

**Table 3:** Comparisons between the groups for inflammatory marker, FFA, and serum adipocytokine levels at baseline and at the final visit (week 16).

Parameters	Randomization visit			Final visit		
	Group 1 Insulin + Ros	Group 2 Insulin only	*P*	Group 1 Insulin + Ros	Group 2 Insulin only	*P*
ESR (mm/h)	11.85 ± 11.36	10.89 ± 10.39	0.969	14.44 ± 13.14	11.00 ± 8.76	0.599
Fibrinogen (g/L)	4.00 ± 1.08	3.46 ± 0.90	0.047	3.46 ± 0.90	3.11 ± 0.92	0.271
hs-CRP (mg/L)	2.32 ± 2.64	1.41 ± 1.69	0.361	1.76 ± 1.14	1.76 ± 2.31	0.443
FFA (mEq/L)	0.57 ± 0.43	0.49 ± 0.25	0.917	0.53 ± 0.28	0.47 ± 0.26	0.518
Resistin (ng/mL)	6.96 ± 3.06	7.16 ± 2.30	0.489	4.99 ± 2.64	5.57 ± 2.48	0.650
Leptin (ng/mL)	25.8 ± 17.6	16.72 ± 16.1	0.130	20.1 ± 12.55	14.0 ± 13.4	0.724
Adiponectin (*µ*g/mL)	17.48 ± 10.71	11.90 ± 5.23	0.760	19.81 ± 11.21	15.98 ± 9.47	0.091

Ros: rosiglitazone; ESR: erythrocyte sedimentation rate; hs-CRP: high-sensitivity C-reactive protein; FFA: free fatty acids.

**Table 4:** Complications observed in the groups.

Complication	Group 1 Insulin + Ros	Group 2 Insulin	*P*
Minor hypoglycemia events experienced per patient	5.35	4.61	0.437
Major hypoglycemia events	2	—	NA
Acute metabolic complication DK/DKA/NKNAHS	—	2	NA
Mean weight gain (kg)	2.58 ± 3.10	1.47 ± 1.53	0.142

Ros: rosiglitazone; DK: diabetic ketosis; DKA: diabetic ketoacidosis; NKNAHS: nonketotic nonacidotic hyperosmolar syndrome; NA: not available.
